# Comorbidity Count Versus the Charlson Comorbidity Index in Predicting Hospital Stay After Hip Fracture in Older Adults: A Retrospective Cohort Study

**DOI:** 10.3390/medicina62101931

**Published:** 2026-10-07

**Authors:** Irina Sîrbu, Anca Iuliana Pîslaru, Adina-Carmen Ilie, Ramona Ștefăniu, Sabinne-Marie Albișteanu, Ana-Maria Turcu, Bogdan Veliceasa, Bogdan Puha, Ioana Dana Alexa, Manuela Poroh

**Affiliations:** 1 Orthopaedic and Traumatology Clinic, “Sf. Spiridon” Clinical Emergency Hospital, 700111 Iasi, Romania; sirbu_irina@d.umfiasi.ro (I.S.); bogdan.veliceasa@umfiasi.ro (B.V.); bogdan.puha@umfiasi.ro (B.P.); 2 Department of Orthopedics and Traumatology, Faculty of Medicine, “Grigore T. Popa” University of Medicine and Pharmacy, 700115 Iasi, Romania; 3 Department of Medical Specialties II, Faculty of Medicine, University of Medicine and Pharmacy “Grigore T. Popa”, 700115 Iasi, Romania; adina.ilie@umfiasi.ro (A.-C.I.); ramona.stefaniu@umfiasi.ro (R.Ș.); sabinne-marie.taranu@umfiasi.ro (S.-M.A.); ana-maria_turcu@umfiasi.ro (A.-M.T.); ioana.alexa@umfiasi.ro (I.D.A.); 4 Department of Biomedical Sciences, Faculty of Medical Bioengineering, “Grigore T. Popa” University of Medicine and Pharmacy of Iasi, 700454 Iasi, Romania; manuela-gabi.poroh@umfiasi.ro

**Keywords:** older adults, hip fracture, length of stay, comorbidity, Charlson comorbidity index, time to surgery

## Abstract

*Background and Objectives*: Hip fracture in older adults is associated with high morbidity, and length of hospital stay is the simplest summary of the acute admission. We aimed to identify the clinical factors associated with prolonged hospital stay in older adults admitted with a hip fracture, and to test whether a weighted comorbidity index predicts stay better than a simple count. *Materials and Methods*: A retrospective cohort of 624 consecutive patients aged 65 years and older with a trochanteric or femoral neck fracture was studied. Length of stay (LOS) was strongly right-skewed (skewness 5.02) and was log-transformed; comparisons used Mann–Whitney and Kruskal–Wallis tests and Spearman correlations, with multivariable models fitted on 487 operated patients with complete data. Postoperative stay was analyzed as a secondary outcome because the preoperative interval forms part of the total stay. *Results*: Median stay was 10 days (IQR 8–13) and median wait for surgery was 4 days (IQR 2–6), with only 25.2% operated on within 48 h. In-hospital mortality was 5.3%. The number of comorbidities was the only patient characteristic consistently associated with stay (rho = 0.119, *p* = 0.003; 1.4% per comorbidity, *p* < 0.001). The Charlson Comorbidity Index (CCI) showed no association in the unweighted, age-adjusted or severity-graded form. Age, sex and admission hemoglobin effects did not survive non-parametric testing. *Conclusions*: A simple count of active comorbidities was associated with LOS whereas the CCI was not, and the model containing the count fitted modestly better (adjusted R^2^ 0.385 vs. 0.365; index term non-significant). This hypothesis-generating finding suggests that the comorbidity count, which is available on admission, may serve as a pragmatic screening tool for selecting patients for comprehensive geriatric assessment; its use in place of the CCI would require validation in independent cohorts. The preoperative wait remains the dominant modifiable target, although its effect on total stay is largely structural.

## 1. Introduction

Hip fracture in an older patient is rarely an isolated injury [1,2,3,4]. It occurs in people whose physiological reserve is already reduced, who usually take several medications and who carry two or more chronic diseases, and it converts a slowly progressing frailty into an emergency that has to be dealt with in hours. The outcomes are correspondingly poor. Many patients never return to their previous walking ability, a substantial proportion are discharged to institutional care, and mortality during the first year after surgery is higher than after most elective orthopedic procedures [1].

Of the whole care pathway, acute admission is the part that the treating team can influence most directly, and length of stay (LOS) is the simplest available summary of it. LOS reflects how ill the patient was on arrival, how quickly the preoperative work-up and the anesthetic assessment were completed, whether complications occurred, and whether a rehabilitation bed or a suitable home was available at discharge. It is also more than a cost item, since every additional day in bed carries its own risk of immobilization, delirium, hospital-acquired infection and further loss of function [5].

Some determinants of LOS are already fixed when the patient reaches the ward: age, sex, the fracture pattern, the burden of chronic disease and the hematological status on admission. Others depend on how the service is organized, above all the interval between admission and surgery and the decision whether to operate at all [6,7]. How much of the variation in LOS belongs to each of these two groups is what tells a department whether it can realistically shorten its stays, or whether its figures mainly reflect the patients it receives.

We therefore examined a consecutive series of older patients admitted with a hip fracture to a single orthopedic department. Our aims were to describe their clinical profile, to identify the factors associated with a prolonged stay, and to establish which of the resulting predictors are actually available early enough to be of use in triage. We also asked whether a weighted comorbidity index, in the form most often recommended for this population, predicts stay better than a simple count of active diagnoses.

Our working hypothesis was that the fracture pattern would matter less than the combination of comorbidity, perioperative anemia and preoperative delay, and that these variables, all available within hours of admission, would be sufficient to identify patients at risk of a long stay.

## 2. Materials and Methods

### 2.1. Study Design and Setting

This is a retrospective observational cohort study conducted in a single orthopedic department. All patients admitted between February 2025 and July 2026 were considered. The study was performed in accordance with the Declaration of Helsinki Ethical Principles for Medical Research and was approved by the Ethics Committee of the “Sf. Spiridon” Clinical Emergency Hospital, Iasi, Romania (approval no. 48, 18 February 2025). Because the analysis was based exclusively on anonymized data retrieved retrospectively from the clinical records of the index admission, no intervention was performed on the patients and no deviation from standard care occurred.

### 2.2. Study Population

**Figure 1:**
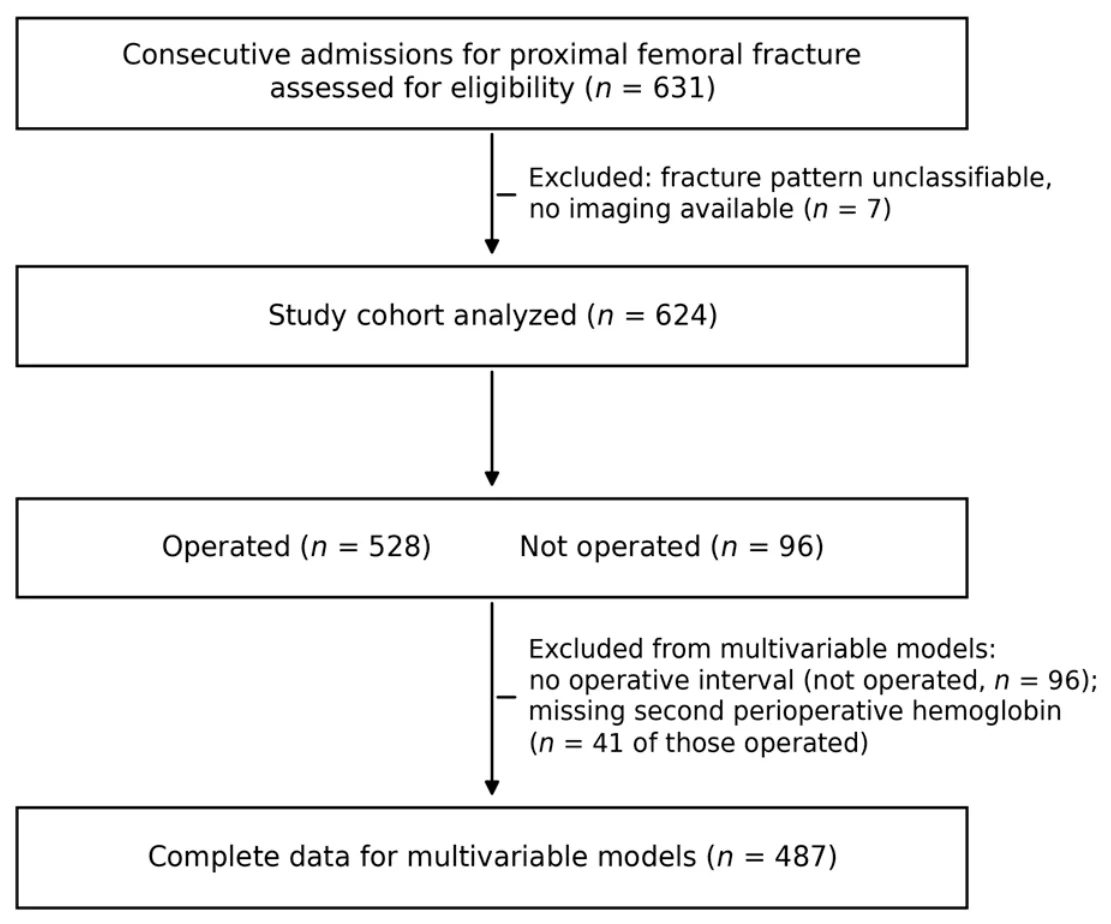
Participant flow through the study, reported according to STROBE. Arrows indicate the sequence of inclusion steps, with exclusions shown beside each arrow; n, number of patients.

The study group comprised 624 consecutive patients aged 65 years and older admitted with a low-energy proximal femoral fracture. Two fracture patterns were included: trochanteric fracture of the femur and femoral neck fracture. Patients were identified from the departmental admission records, which held 631 consecutive admissions between February 2025 and July 2026; the seven records without a documented fracture pattern were excluded, resulting in the 624 patients analyzed here. In these cases, no radiographic examination was retrievable from the index admission, so the fracture could not be classified as femoral neck or trochanteric; they represented 1.1% of assessed admissions, a proportion too small to have materially affected the estimates reported below. Of these, 528 underwent surgery and 487 had complete data for every variable entered in the multivariable models (Figure 1). Each patient was followed through the electronic and paper record of the index admission until discharge or in-hospital death. No patient was excluded on the basis of comorbidity, functional status, or the decision not to operate; the non-operated patients were retained in the cohort and analyzed as a separate management category.

### 2.3. Variables and Data Collection

The dependent variable was the total length of hospital stay (LOS), recorded in days from admission to discharge or death.

The independent variables were: age (years); sex; area of residence (urban or rural); fracture type; number of comorbidities; individual comorbidity categories (cardiovascular, diabetes mellitus, neurological, psychiatric, anemic syndrome, hepatic, renal, malignancy, and obesity); hemoglobin concentration at admission (HbT1) and at the second perioperative determination (HbT2); the interval in days between admission and surgery; whether a surgical intervention was performed; the need for transfusion; and death, recorded separately as postoperative death and as death without surgery (in-hospital death in non-operated patients). Mortality was analyzed with the explicit caveat that death may truncate the recorded stay and thereby shorten LOS artificially.

Comorbidity burden was quantified in two ways. First, the unweighted count of comorbidity categories present at admission was recorded for each patient. Second, the Charlson Comorbidity Index (CCI) was computed by applying the standard Charlson weights [8] to the comorbidity categories available in the database: one point each for diabetes mellitus, cardiovascular disease, neurological disease, and liver disease, and two points each for renal disease and malignancy. Psychiatric disorders, anemic syndrome, and obesity are not components of the CCI and were therefore weighted zero. An age-adjusted Charlson Comorbidity Index (ACCI) was derived by adding one point for each decade of age from 50 years onwards, giving four additional points to patients aged 80 years and older.

Because the database records comorbidities as broad categories, the unweighted CCI score cannot by itself distinguish uncomplicated from end-organ disease. We therefore derived a severity-graded version of the index from the admission laboratory data. Estimated glomerular filtration rate (eGFR) was calculated from admission creatinine, age, and sex using the race-free 2021 CKD-EPI equation [9]; renal disease scored two points only when eGFR fell below 30 mL/min/1.73 m^2^, or when a recorded renal diagnosis coincided with an eGFR below 60; and diabetes mellitus was scored two points, as diabetes with chronic complications, when accompanied by an eGFR below 60 and one point otherwise. Because this grading rests on a single creatinine value obtained at admission in an acutely injured patient, it cannot separate chronic kidney disease from acute kidney injury, and the graded index should be read as an approximation on that basis. Anemia at admission was graded on the World Health Organization thresholds (mild, moderate, or severe, by sex) [10]. In addition, an acute laboratory derangement score was constructed as the count of eight abnormalities present at admission: hyponatremia, hypernatremia, hypokalemia, hyperkalemia, urea above 60 mg/dL, glycemia above 140 mg/dL, eGFR below 60 mL/min/1.73 m^2^, and moderate-to-severe anemia.

The CCI and its derivatives were computed for all 624 patients. Multivariable models also required the laboratory variables and the interval to surgery, and were therefore fitted on the 487 patients with complete data for every predictor. Missing values arose almost entirely from two sources: the second perioperative hemoglobin determination was absent in 106 of 624 records (17.0%), and the interval to surgery was undefined for the 96 patients who were not operated on. Because the interval to surgery cannot exist without an operation, every multivariable model reported here describes patients who underwent an operation only, and no imputation was attempted. All comparisons between comorbidity measures were made on that same common sample so that the fit statistics are directly comparable.

### 2.4. Statistical Analysis

Data were entered and analyzed in SPSS Statistics version 18.0 (SPSS Inc., Chicago, IL, USA). Continuous variables are reported as the median with interquartile range (IQR) and minimum–maximum, and additionally as the mean ± standard deviation where comparison with earlier reports requires it; categorical variables are reported as absolute frequency and percentage. The significance level was set at 5% (two-sided).

LOS was far from normally distributed (skewness 5.02; kurtosis 49.92), so parametric analysis on the original scale was not defensible. We log-transformed the variable for all regression work, which brought it within conventional limits (skewness −0.67; kurtosis 2.65). Group comparisons were made with the Mann–Whitney U test. Levene’s test was used to check equality of variances, and Welch’s t-test is reported beside each comparison as a sensitivity analysis; the one-way ANOVA results from the untransformed scale are also given, so that they can be set against the earlier literature. Comparisons across the three CCI strata used the Kruskal–Wallis test, with ANOVA used alongside.

Given the skew of the outcome, correlations between LOS and the continuous variables were quantified primarily with Spearman’s rank correlation coefficient, with the Pearson coefficient reported alongside this for comparability with previous reports. Coefficient values lie within the interval [−1, +1]: values approaching ±1 indicate a strong monotonic association and values approaching 0 indicate a weak one.

Independent predictors were identified by multivariable linear regression on log-transformed LOS. The five predictors were pre-specified from the study hypothesis (age, HbT1, HbT2, number of comorbidities, and interval to surgery) and entered sequentially in that order, so that the incremental contribution of each could be read from the change in R^2^. We report, for each step, R, R^2^, adjusted R^2^, the standard error of the estimate, the F statistic and *p* value of the whole model, and separately, the change in R^2^ with its F change and *p* value; the two F statistics are distinguished because they are frequently confused. Regression coefficients on the log scale are reported both as coefficients and as the corresponding percentage change in LOS. No automated stepwise selection was used, since data-driven selection inflates R^2^ and invalidates the nominal *p* values.

Because the interval to surgery is by definition part of the total LOS, any association between the two is partly structural. To separate the two effects, all models were repeated with postoperative LOS (total stay minus the preoperative interval) as the dependent variable; this quantity measures recovery and discharge time and is mathematically independent of the waiting interval. To determine whether a weighted or severity-graded comorbidity score outperforms a simple count, otherwise identical models were fitted on the same complete-case sample, each containing one of five candidate measures: the unweighted number of comorbidities, the CCI, the ACCI, the severity-graded index, and eGFR; the acute laboratory derangement score was tested in the same way. Because the ACCI is by construction the sum of the CCI score and a function of age, it was entered together with age only for the explicit comparison of comorbidity measures, where the resulting collinearity is acknowledged.

No a priori sample size calculation was performed, since the study analyzed all consecutive eligible admissions in the study period. Post hoc, a complete-case sample of 487 with five predictors provides more than 90% power to detect an incremental R^2^ of 0.03 at a two-sided alpha of 0.05, and approximately 80% power for an incremental R^2^ of about 0.015. All analyses are exploratory: multiple correlations and group comparisons were examined without correction for multiplicity, so individual *p* values close to 0.05 should be read as hypothesis-generating rather than confirmatory. Claude (Anthropic, San Francisco, CA, USA) was used for language editing of the manuscript and for checking the consistency of the reported statistics against the tables; it was not used for data collection, statistical analysis or interpretation of the results.

## 3. Results

### 3.1. Characteristics of the Study Group

The cohort comprised 624 patients. Women accounted for 451 cases (72.3%) and men accounted for 173 (27.7%). The mean age was 79.73 ± 7.79 years, with a median of 80 years and a range of 65 to 101 years; the cohort was almost exactly halved by the 80-year threshold, with 307 patients (49.2%) below and 317 (50.8%) at or above that age. Residence was similarly balanced between urban (321; 51.4%) and rural (303; 48.6%) settings. Trochanteric fractures of the femur were the more frequent pattern (396; 63.5%), followed by femoral neck fractures (228; 36.5%).

The CCI averaged 1.68 ± 1.19 points (median 1; range 0–7), and 149 patients (23.9%) scored three points or more. The ACCI averaged 5.07 ± 1.40 points (median 5; range 2–11), reflecting the age structure of the cohort rather than its organ-specific disease burden. The severity-graded index averaged 1.63 ± 1.20 points (median 1; range 0–6) (Table 1).

Comorbidity was near-universal. Cardiovascular disease was present in 542 patients (86.9%), followed by anemic syndrome in 209 (33.5%), psychiatric disorders in 124 (19.9%), renal disease in 107 (17.1%), neurological disease in 102 (16.3%), and diabetes mellitus in 100 (16.0%); malignancy (34; 5.4%), obesity (40; 6.4%) and hepatic disease (23; 3.7%) were less common. Surgical stabilization was performed in 528 patients (84.6%) and transfusion was required in 12 patients (1.9%). Median LOS was 10 days (IQR 8–13; range 1–95) and median postoperative stay among operated patients was 6 days (IQR 5–7). Thirty-three patients died during the index admission, reaching an in-hospital mortality of 5.3%; these comprised 22 deaths after surgery (3.5% of the cohort and 4.2% of those operated on) and 11 deaths in patients who were never operated on (1.8% of the cohort), with the two categories being mutually exclusive (Table 1).

Waiting times for surgery were long. Among the 528 operated patients the median interval was 4 days (IQR 2–6; range 0–18). Thirty-three patients (6.2%) reached theater within one calendar day and 133 (25.2%) within two days, while 191 (36.2%) waited three or four days and 204 (38.6%) waited longer than four days. Fewer than one in four patients met the 48 h target used by most national hip-fracture programs.

### 3.2. Univariate Analysis of Length of Stay

Median LOS for the whole cohort was 10 days (IQR 8–13), with a mean of 10.62 ± 6.56 days. Only one of the comparisons tested was robust to the skewness of outcomes. Operated patients stayed markedly longer than those managed non-operatively (median 10 days, IQR 8–13, vs. 4 days, IQR 3–6; Mann–Whitney *p* < 0.001; Welch *p* < 0.001), and patients who died after surgery had the longest stays in the series (median 17.5 days, IQR 13–25, vs. 10 days, IQR 8–12; Mann–Whitney *p* < 0.001; Welch *p* < 0.001).

The apparent difference between sexes did not survive proper testing. Men and women had the same median stay (10 days in both; IQR 7–13 vs. 8–12), the variances differed markedly (SD 9.49 vs. 4.98; Levene’s *p* < 0.001), and neither the Mann–Whitney test (*p* = 0.514) nor Welch’s t-test (*p* = 0.129) showed a difference; the nominally significant one-way ANOVA result on the untransformed scale (*p* = 0.049) is an artifact of the variance inequality and the extreme right tail in men, whose maximum stay reached 95 days compared to 55 days in women. LOS was likewise independent of area of residence (median 10 days in both; Mann–Whitney *p* = 0.396; ANOVA *p* = 0.879) and fracture type (median 10 days in both; Mann–Whitney *p* = 0.835; ANOVA *p* = 0.843). Death without surgery was associated with a shorter stay (median 6 days, IQR 4–10, vs. 10 days, IQR 8–13; Mann–Whitney *p* = 0.045; Welch *p* = 0.060), a pattern consistent with early death truncating the admission rather than with a protective effect (Table 2).

### 3.3. Correlations with Continuous Variables

**Figure 2:**
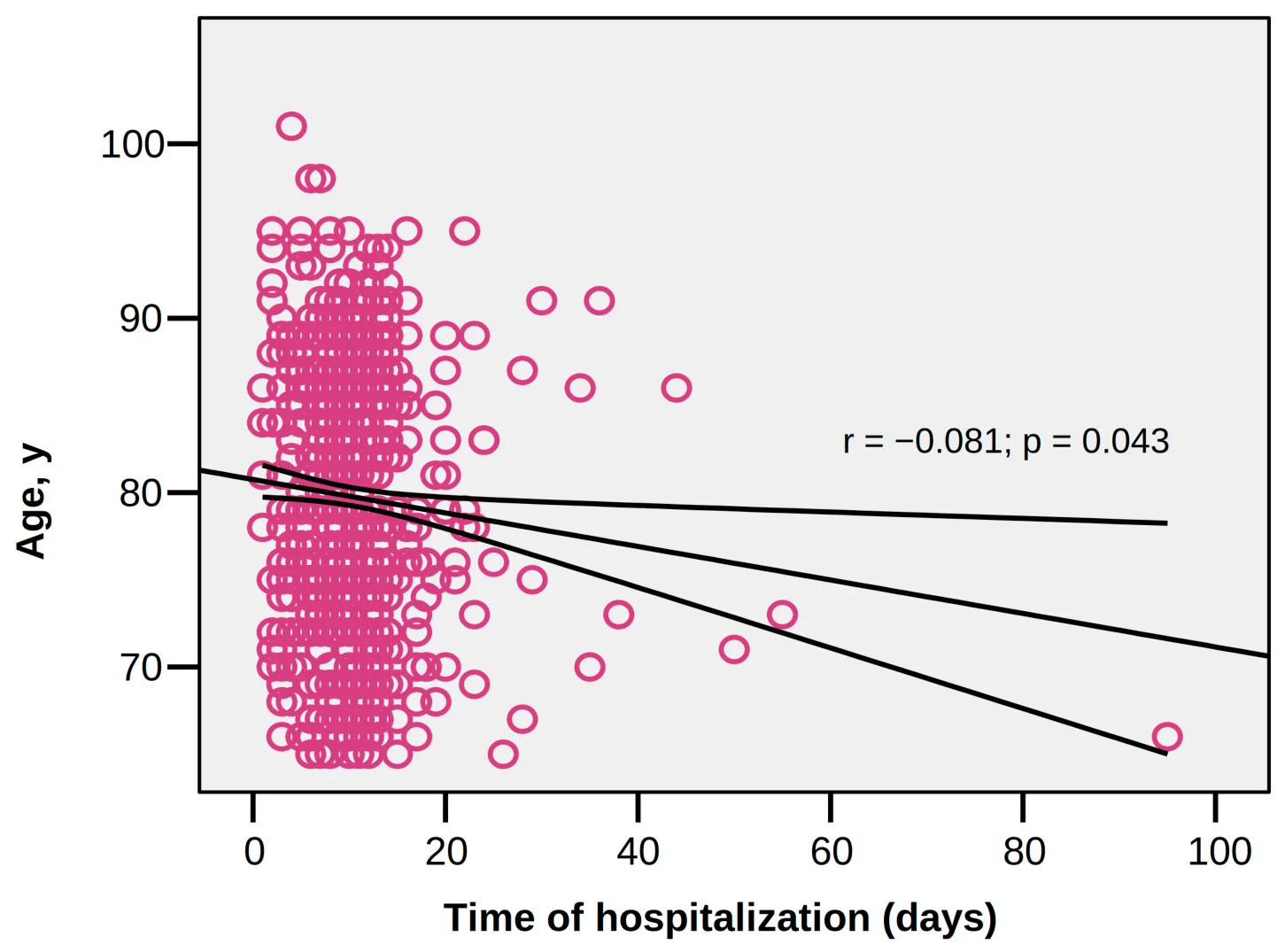
Age and length of hospital stay. The coefficient shown on the plot is the Pearson correlation (r = −0.081; *p* = 0.043); on the rank test, the association was not significant (Spearman’s rho = −0.036; *p* = 0.371). Circles represent individual patients; the central line is the linear regression fit and the outer lines its 95% confidence band.

**Figure 3:**
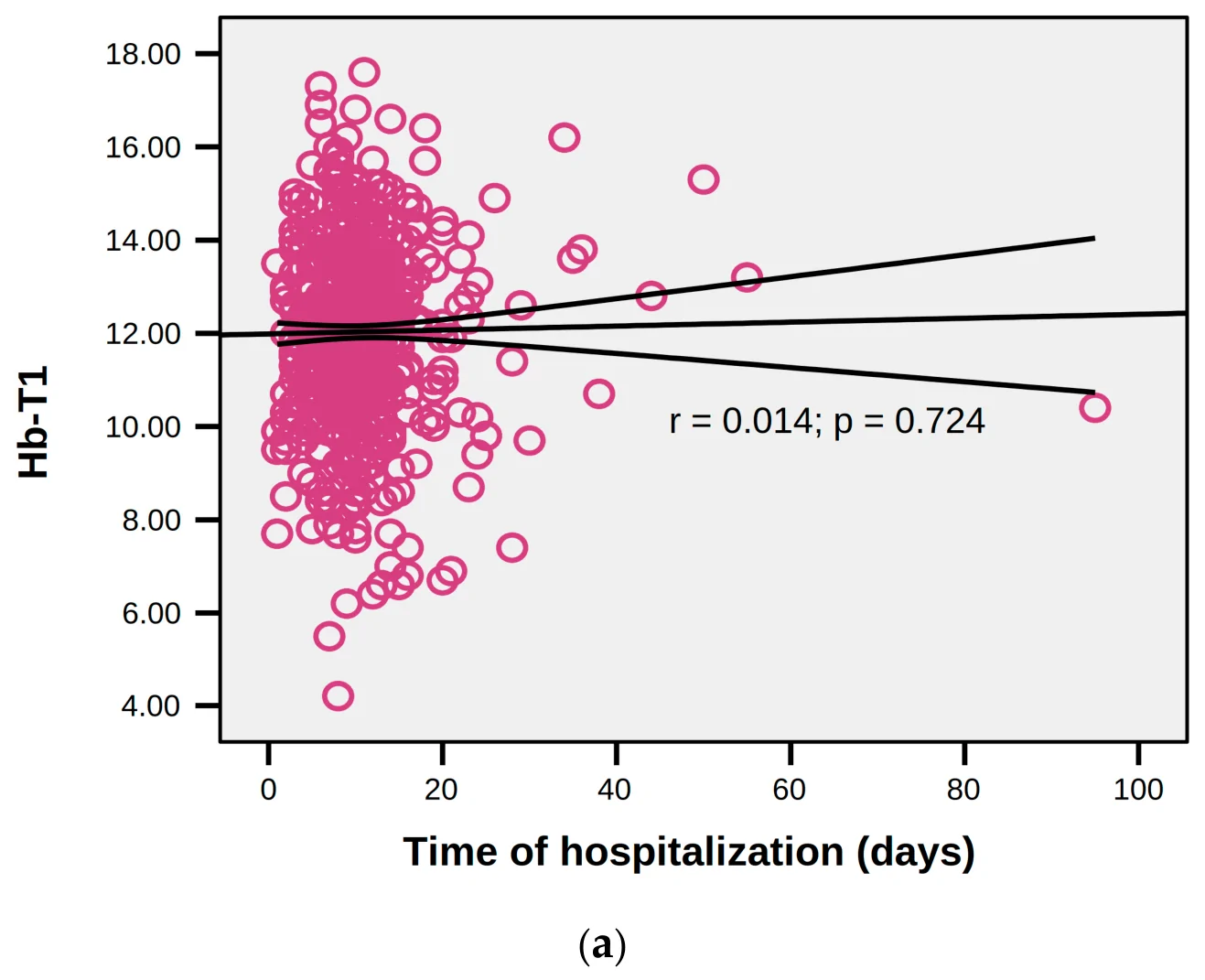

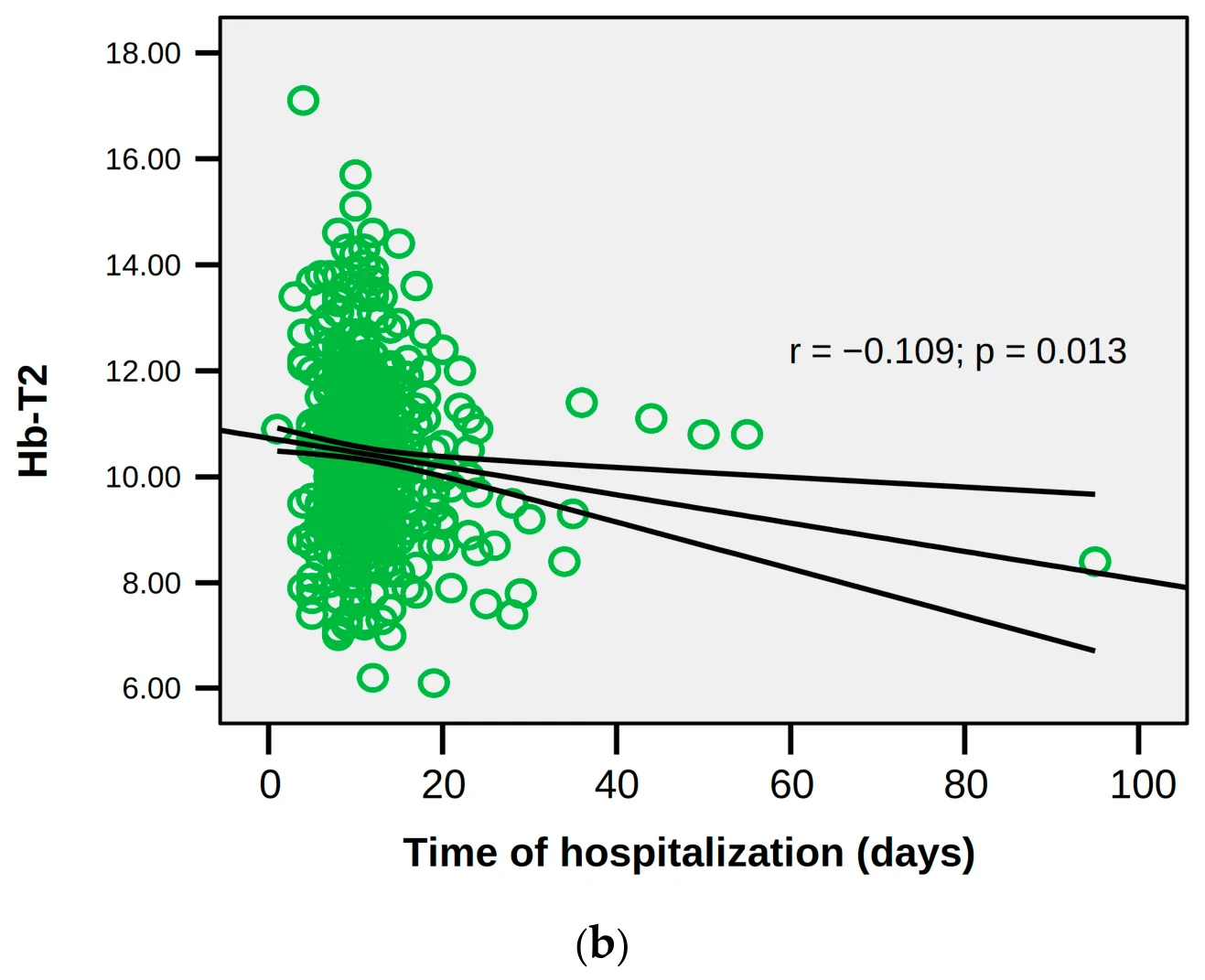
Perioperative hemoglobin and length of hospital stay: (**a**) hemoglobin at admission, HbT1 (Pearson’s r = 0.014; *p* = 0.724); (**b**) hemoglobin at the second perioperative determination, HbT2 (Pearson’s r = −0.109; *p* = 0.013). Neither association was significant on the rank test (Spearman’s rho = 0.038 and −0.064). Circles represent individual patients; the central line is the linear regression fit and the outer lines its 95% confidence band.

**Figure 4:**
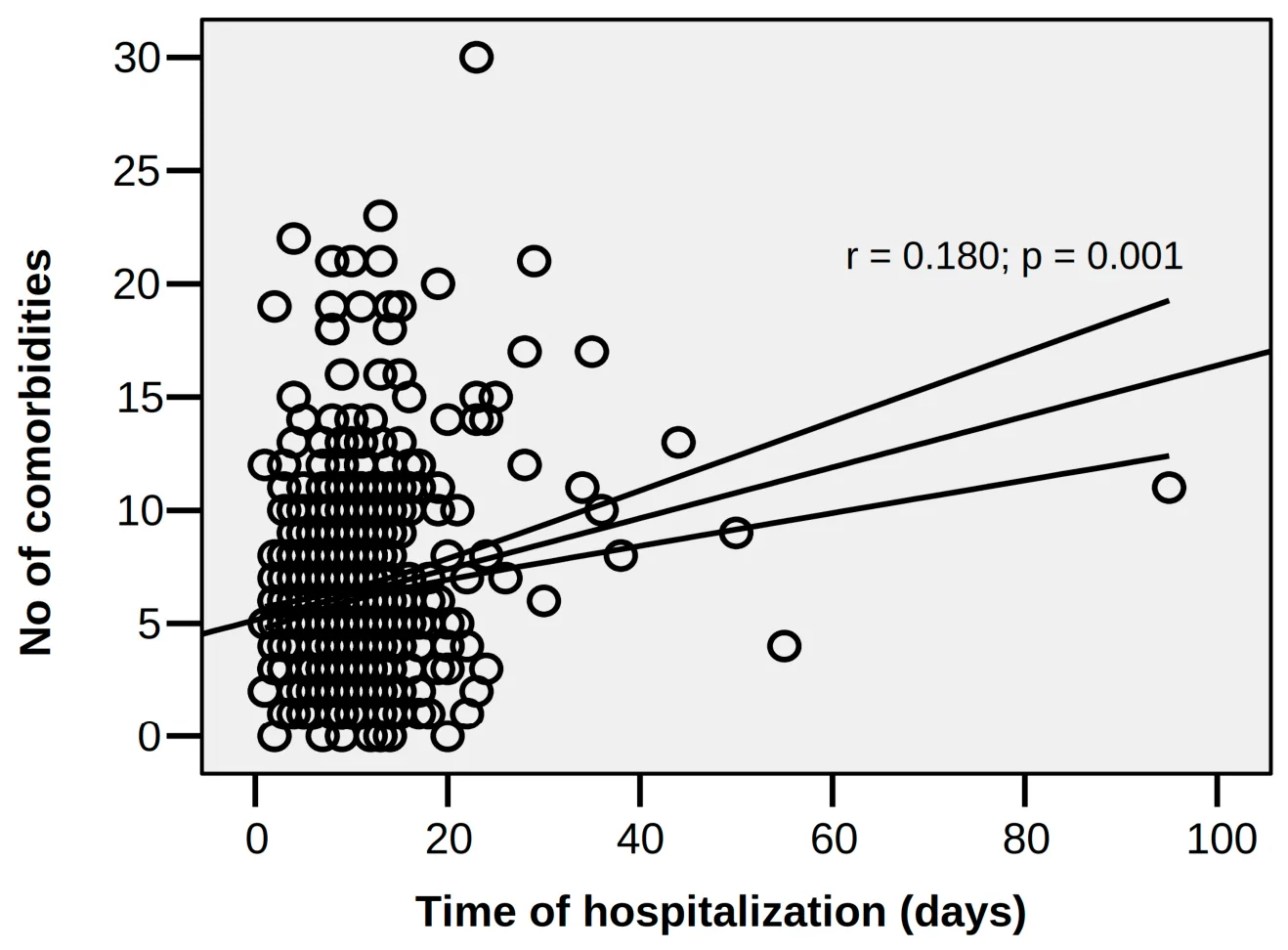
Number of comorbidities and length of hospital stay (Pearson’s r = 0.180; Spearman’s rho = 0.119; *p* = 0.003). Circles represent individual patients; the central line is the linear regression fit and the outer lines its 95% confidence band.

**Figure 5:**
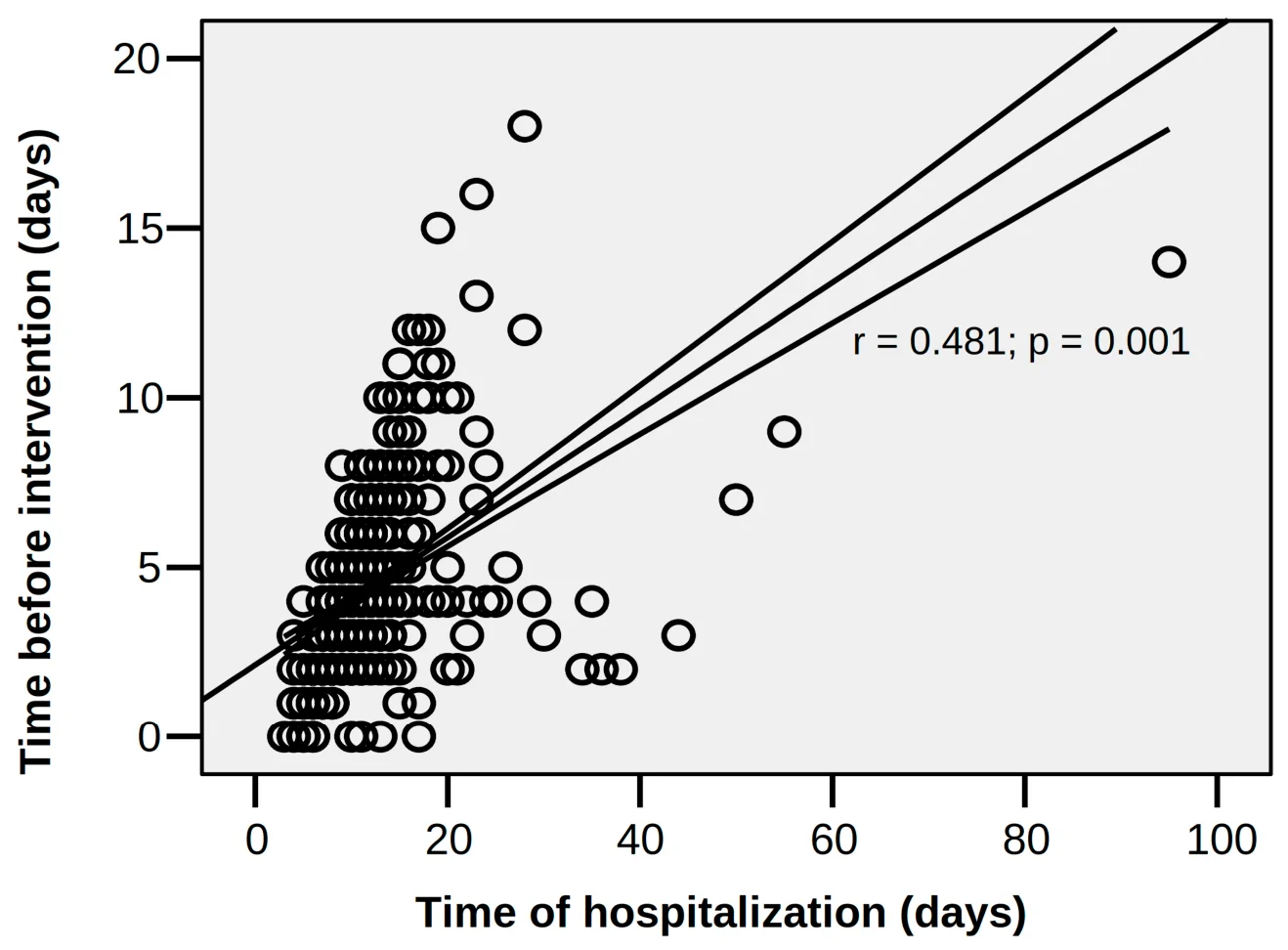
Interval from admission to surgery and length of hospital stay (Pearson’s r = 0.481; Spearman’s rho = 0.670; *p* < 0.001). Part of this association is structural, since the waiting interval is a component of the total stay. Circles represent individual patients; the central line is the linear regression fit and the outer lines its 95% confidence band.

Once the analysis was considered on a rank basis, only two variables remained associated with LOS: the interval to surgery (Spearman’s rho = 0.670; *p* < 0.001; Pearson’s r = 0.481) and the number of comorbidities (rho = 0.119; *p* = 0.003; Pearson’s r = 0.180). Three variables that were significant on the Pearson coefficient were not significant on the rank test: age (rho = −0.036; *p* = 0.371 vs. r = −0.081; *p* = 0.043), hemoglobin at admission (rho = 0.038; *p* = 0.348 vs. r = 0.014; *p* = 0.724) and the second perioperative determination (rho = −0.064; *p* = 0.143 vs. r = −0.109; *p* = 0.013). The long right tail of the outcome accounts for the difference, and we therefore treat the associations of age and hemoglobin as unconfirmed (Figure 2, Figure 3, Figure 4 and Figure 5).

The weighted comorbidity score differed from the simple count. Neither the CCI (rho = −0.032; *p* = 0.418; Pearson’s r = −0.004; *p* = 0.929) nor the ACCI (rho = −0.028; *p* = 0.491; Pearson’s r = −0.043; *p* = 0.285) showed any association with LOS, even though the index correlated strongly with the raw comorbidity count (r = 0.508; *p* < 0.001). Weighting the comorbidities thus removed the signal carried by their number rather than sharpening it.

Part of the association between waiting time and total stay is structural, since the waiting days are themselves counted in the stay. Repeating the analysis against postoperative LOS, which cannot contain the waiting interval, resulted in almost nothing: Spearman’s rho = 0.034 (*p* = 0.440), Pearson’s r = 0.102 (*p* = 0.020). The number of comorbidities, by contrast, remained associated with postoperative stay (rho = 0.165; *p* < 0.001). Delay lengthens the admission chiefly by adding the days spent waiting; what happens afterward changes little.

### 3.4. Multivariable Analysis

**Figure 6:**
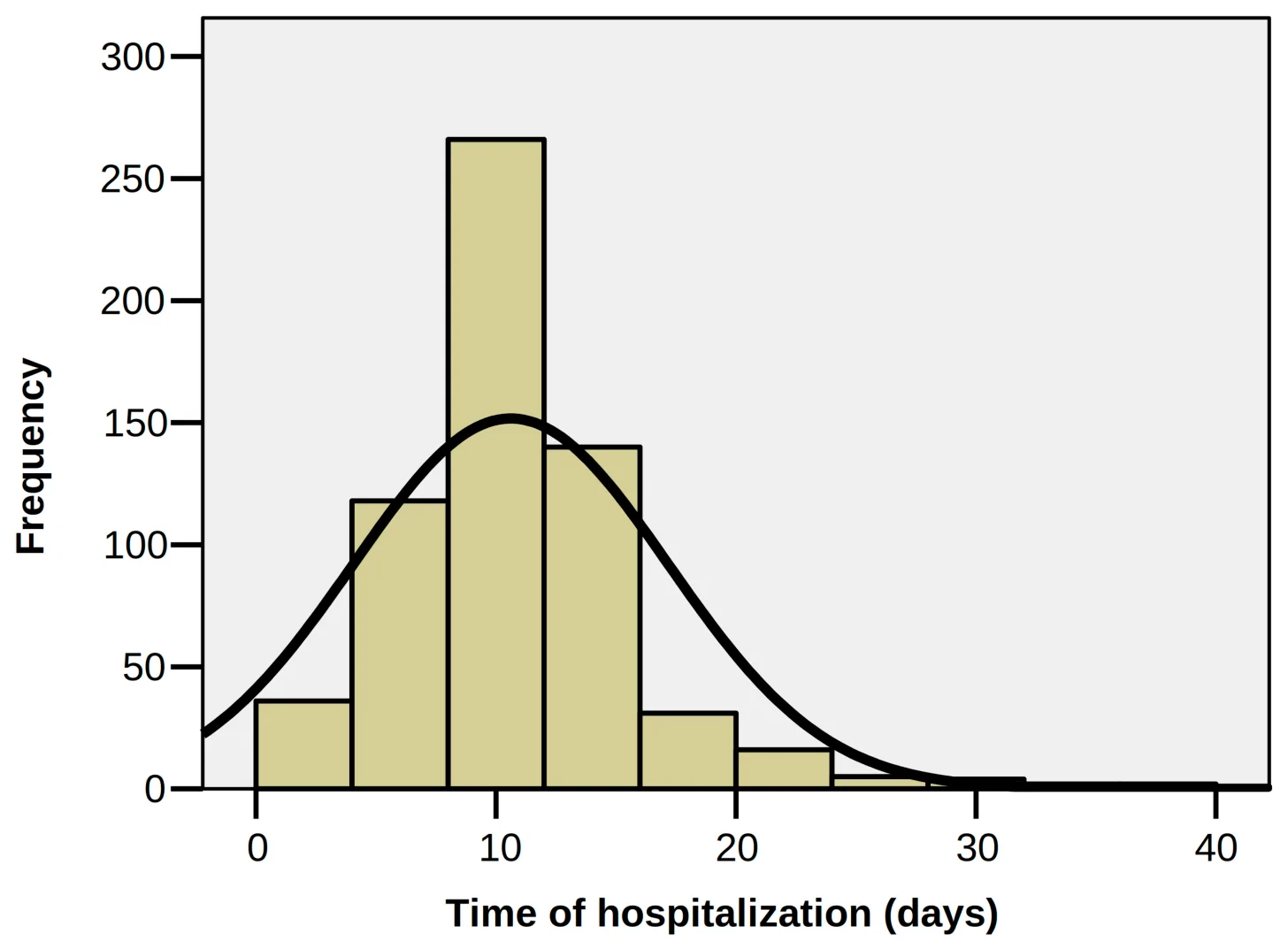
Distribution of length of hospital stay in the study group (*n* = 624; median 10 days, IQR 8–13, range 1–95). The distribution is markedly right-skewed (skewness 5.02; kurtosis 49.92), which is why all regression modeling was performed on the log-transformed outcome. Bars show the observed frequency distribution and the curve is the fitted normal distribution.

Because LOS was strongly right-skewed (skewness 5.02), all models were fitted on the log-transformed outcome, for which the distribution was acceptable (skewness −0.67) (Figure 6). The five pre-specified predictors were age, HbT1, HbT2, the number of comorbidities and the interval to surgery, entered in that order for the 487 operated patients with complete data.

Age alone explained about 1% of the variance in log LOS (Model 1: R^2^ = 0.012; adjusted R^2^ = 0.010; F = 5.78; *p* = 0.017), and hemoglobin at admission added nothing (Model 2: change in R^2^ = 0.001; *p* = 0.436). The second hemoglobin determination (Model 3: change in R^2^ = 0.009; *p* = 0.034) and the number of comorbidities (Model 4: change in R^2^ = 0.058; *p* < 0.001) each contributed to this, and the interval to surgery produced by far the largest increment (Model 5: change in R^2^ = 0.312; *p* < 0.001). The full model reached R = 0.626, R^2^ = 0.391 and adjusted R^2^ = 0.385 (F = 61.89; *p* < 0.001), explaining 38.5% of the variance in log LOS (Table 3).

On the log scale the coefficients translate into multiplicative effects: each additional day of preoperative delay was associated with an 8.8% longer total stay (b = 0.0843; *p* < 0.001); each additional comorbidity was associated with a 1.4% longer stay (b = 0.0137; *p* < 0.001); each g/dL of admission hemoglobin was associated with a 1.9% longer stay (b = 0.0193; *p* = 0.015); and each g/dL of the second determination was associated with a 2.2% shorter stay (b = −0.0227; *p* = 0.024). Age was not significant (b = −0.0014; *p* = 0.453). When the same model was fitted to postoperative LOS, the explained variance fell to 4.9% (R = 0.242; adjusted R^2^ = 0.049; F = 6.00; *p* < 0.001) and the interval to surgery was no longer significant (b = 0.149 days per day of delay; *p* = 0.145), whereas the number of comorbidities remained so (b = 0.238 days per comorbidity; *p* < 0.001) as did the second hemoglobin determination (b = −0.461; *p* = 0.016). The apparent dominance of preoperative delay in the total-stay model is therefore largely arithmetic: a coefficient of 1.0 would mean that a day of waiting adds exactly one day to the admission and nothing more, and the estimate obtained here (1.149 days on the untransformed scale) implies only about 0.15 additional days of postoperative stay per day of delay, an increment that did not reach significance.

### 3.5. Comorbidity Count Versus Weighted Comorbidity Index

LOS did not differ across CCI strata: median 10 days (IQR 8–12.75) for a score of 0–1 (*n* = 362); 10 days (IQR 7–12) for 2–3 (*n* = 208); and 11 days (IQR 8–13) for 4 or more (*n* = 54, Kruskal–Wallis *p* = 0.169; one-way ANOVA *p* = 0.184). The relationship was not monotonic, and the intermediate stratum had the lowest median (Table 4).

Substituting the weighted index for the unweighted count slightly reduced rather than improved the fit. Of the 487 patients with complete data, the model containing the number of comorbidities reached an adjusted R^2^ of 0.385 with a significant comorbidity term (b = 0.0137; *p* < 0.001). Replacing it with the CCI lowered the adjusted R^2^ to 0.365 and left the index term non-significant (b = 0.0056; *p* = 0.623), and the ACCI was identical (adjusted R^2^ = 0.365; b = 0.0072; *p* = 0.516). The same ordering held for postoperative LOS, where the count retained significance (b = 0.238; *p* < 0.001) while the index did not (b = 0.102; *p* = 0.639).

Grading the index by severity did not recover the lost signal. The estimated glomerular filtration rate had a median of 76.1 mL/min/1.73 m^2^, with 28.4% of patients below 60 and 4.6% below 30, so there was an adequate severity gradient to work with. The graded index nevertheless correlated no better with LOS than the unweighted one (rho = −0.031; *p* = 0.432), and neither did eGFR alone (rho = −0.064; *p* = 0.113), the World Health Organization anemia grade (rho = −0.038; *p* = 0.350), or the acute laboratory derangement score (rho = 0.043; *p* = 0.289). In the multivariable models each of these measures produced an adjusted R^2^ between 0.365 and 0.367, which is below the 0.385 obtained with the plain count, and the comorbidity term was not significant in any of them. When the graded index was entered alongside the count, the count remained significant and the index did not, so the weighting added no information beyond the number of active diagnoses (Table 5).

## 4. Discussion

What stands out in this cohort is how long patients waited for their operation. The median interval was 4 days, a quarter of the patients reached theater within 48 h, and more than a third waited longer than four days. This was measured against the 24 to 48 h standard of national hip-fracture programs, which is a considerable organizational shortfall, and it is the finding with the clearest bearing on practice. However, the effect on LOS has to be stated carefully. Waiting time is part of admission, so the strong association with total stay (rho = 0.670) is largely arithmetic. Set against postoperative stay, which includes no waiting days, the association nearly disappears (rho = 0.034), and in the multivariable model, the interval was no longer significant, adding only about 0.15 postoperative days per day of delay (Section 3.4). The argument for operating sooner in this service therefore rests on the waiting days themselves, which are real bed-days and are avoidable, and on the wider evidence that delay raises complications and mortality [11,12,13,14], not on any demonstrated prolongation of recovery in our own data.

The length of the preoperative interval deserves comment in its own right, because it is the finding most open to organizational remedy. The retrospective design did not allow us to document the reason for delay in each patient, but the pattern we observed is characteristic of services in which hip-fracture patients share emergency theater capacity with other trauma cases and with elective work, in which preoperative medical optimization and anesthetic clearance are obtained through sequential specialist consultations rather than from a dedicated team, and in which operating lists are not routinely available at weekends; in a population with near-universal comorbidity, the management of anticoagulant and antiplatelet therapy and the correction of anemia and electrolyte derangements add further hours or days. Long preoperative stays of this kind are not unique to our setting. In a prospective cohort from Belgrade, Tulic et al. reported a mean preoperative stay of 8.4 days and found that a delay beyond 72 h was an independent predictor of general complications and of poorer functional recovery at four months, with every outcome worsening progressively after the fifth day [15]. In a recent Brazilian cohort, the fracture-to-surgery interval was again independently associated with a longer LOS, and the authors pointed to system-level contributors such as access barriers, transport and bed availability rather than to patient factors alone [16]. The organizational response most consistently supported by the literature is orthogeriatric co-management within an integrated care pathway. Mazzola et al. showed that a co-managed program in which the geriatrician acts as the primary attendant, whether started in the emergency department or after surgery, produced satisfactory outcomes in frail patients, while identifying coordination between hospital and rehabilitation services as the aspect most in need of improvement [17]. A recent Swiss study found that an orthogeriatric fast-track pathway that started in the emergency department, combining early multidisciplinary assessment, rapid diagnostics and regional anesthesia, shortened the emergency-department stay and was associated with lower early mortality [18]; meta-analytic evidence indicates that orthogeriatric models shorten time to surgery and LOS and reduce in-hospital complications and mortality [19], and the same has been reported from Spanish and British units [20,21]. In France, orthogeriatric reorganization brought the average stay down to 9–10 days [22], and in the United Kingdom, replacing a geriatric consultation service with a fully integrated orthogeriatric ward reduced the mean time to surgery from 41.8 to 27.2 h and the mean stay from 27.5 to 21 days [23]. Geriatric co-management was not part of the routine pathway in our department during the study period, and the introduction of geriatric involvement from admission, with agreed protocols for anticoagulant management and medical optimization and with protected trauma theater time, would be the logical response to the delays documented here; the comorbidity count could serve as a simple entry criterion for such a pathway.

Comorbidity was more consistent than any other patient characteristic. The number of comorbidities was the only factor that was retained with every specification we tried. It correlated with total stay (rho = 0.119; *p* = 0.003) and postoperative stay (rho = 0.165; *p* < 0.001), and it held its significance in both multivariable models: a 1.4% longer stay per comorbidity on the log scale (*p* < 0.001), and 0.238 additional postoperative days per comorbidity (*p* < 0.001). That so crude a measure carries any signal is itself notable, given that 86.9% of these patients had cardiovascular disease and the count treats well-controlled hypertension and decompensated heart failure alike.

We expected a weighted index to correct this, and it did not. The CCI showed no association with LOS on either coefficient (rho = −0.032; *p* = 0.418); stay did not differ across CCI strata (Kruskal–Wallis *p* = 0.169); and replacing the count with the index lowered the adjusted R^2^ of the log-stay model from 0.385 to 0.365 while leaving the comorbidity term non-significant (*p* = 0.623). Grading the index by severity did not help either: weighting renal disease and diabetes according to the admission glomerular filtration rate performed no better (adjusted R^2^ = 0.366), and neither did eGFR alone, the anemia grade, or a composite score of eight acute laboratory derangements. The same ordering held for postoperative stay. Similar observations have been reported elsewhere. In a linked analysis of 47,698 hip-fracture admissions the index discriminated mortality acceptably but predicted resource use poorly, with adjusted R^2^ values of 0.007 to 0.045 [24], and in a series of lower-extremity trauma in which higher scores prolonged overall stay, no association was found in the hip and pelvis subgroup [25].

A likely explanation is that the CCI was built and validated to predict one-year mortality, so its weights reflect lethality rather than acute perioperative workload; renal disease and malignancy score two points each because they shorten life, not because they add hospital days. Stay in a hip-fracture unit depends on what has to be corrected before an operation can proceed and on what delays discharge afterwards, and the Charlson weights say little about either. The unweighted count performed somewhat better here, perhaps because it treats every active diagnosis as an equal claim on clinical attention, which is closer to the way ward time is spent. We stress, however, that the difference in model fit was modest (0.020 in adjusted R^2^) and was observed in a single retrospective cohort analyzed on a complete-case basis, so it should be read as hypothesis-generating rather than as evidence that one measure is superior to the other. For departments trying to anticipate long stays, a mortality-oriented index may nevertheless be a less suitable instrument than its widespread use would suggest. Where such a score does belong is in prognosis: the CCI predicts 5- and 10-year survival after an operatively treated hip fracture [26,27], and it remains appropriate for that purpose, although even there its discrimination is limited: in a prospective Dutch cohort of 3523 operated patients, the original and the adjusted CCI achieved areas under the curve of only 0.67 to 0.72 for 30-day and 1-year mortality [28].

What follows for practice is limited but concrete. We do not propose the comorbidity count as a replacement for the CCI, whose value in predicting survival is well established, and its use as an alternative measure of acute risk would require validation in independent cohorts and in healthcare settings with a different case mix, theater access and discharge arrangements. What the count offers is availability: it can be read from the admission record within hours of arrival, requires no weighting and no laboratory result, and in our data was the only patient characteristic consistently associated with LOS. It is therefore best regarded as a simple admission-based screening or stratification tool, an initial filter to identify the patients most likely to benefit from comprehensive geriatric assessment, medical optimization and early discharge planning, rather than as a prognostic index in its own right. Stratifying patients at admission by their expected LOS has practical value for bed management and postoperative planning even when the stratifying tool is crude; a recent machine-learning analysis of 3118 operated hip-fracture patients found that first classifying patients into short- and long-stay groups and only then estimating the number of days substantially improved the accuracy of LOS prediction [29]. Whether a comorbidity count threshold can be used in the same way, and whether the patients identified actually benefit from earlier geriatric involvement, are questions for a prospective study.

The hemoglobin findings are weaker than they first appear. Hemoglobin upon admission was unrelated to LOS, whereas the second perioperative determination was inversely related to it on the Pearson coefficient and remained significant in both multivariable models, with the two values entering the regression with opposite signs; this suggests that the size of the perioperative fall matters more than the value on arrival [30,31,32,33]. Because neither correlation survived rank-based testing, we regard the effect of the perioperative fall in hemoglobin as plausible but not established. Incidentally, only 1.9% of patients were transfused, although 33.5% carried a diagnosis of anemic syndrome, which raises the question of whether the transfusion threshold in use was conservative for a population with so little physiological reserve [34].

Age and sex did not show the expected associations. The weak inverse association between age and LOS on the Pearson coefficient disappeared on the rank test and in the multivariable models, and the nominally significant difference between sexes on ANOVA did not survive tests that accounted for grossly unequal variances, with the medians being identical at 10 days; both apparent findings are best treated as artifacts of the skewed distribution. Fracture type and area of residence showed no association on any test. Within a uniformly frail population, then, neither the anatomy of the fracture nor the patient’s demographic profile predicts how long the admission will last; accumulated disease and what the service does about the operation matter more.

The mortality results illustrate a specific analytical problem. Postoperative death was associated with the longest stays in the series (median 17.5 days, mean 18.00 ± 9.15), which fits a complicated postoperative course, whereas death without surgery was associated with shorter stays (median 6 days, mean 7.55 ± 4.85), which fits early death truncating admission before treatment could take its course. LOS in a hip-fracture cohort is thus a competing-risk outcome, and any interpretation that treats death as a neutral censoring factor will be biased.

The risk profile that emerges is a narrow one. Only one variable holds up across every specification: the number of comorbidities on the day of admission. The perioperative fall in hemoglobin is supported by the multivariable models but not by rank correlation, and in any case, it is not available early, since it needs a second determination taken around the time of surgery. The expected interval to surgery is a service-level estimate rather than an individual measurement. Of the five variables in the model, only the comorbidity count is both available within hours of arrival and robust across specifications; hemoglobin upon admission was available early but showed no association with rank correlation and entered the multivariable model with a positive sign, so it cannot be recommended as a triage variable on these data. That leaves a single admission-time trigger. On the log scale, the full model accounts for 38.5% of the variance in total stay but only 4.9% of the variance in postoperative stay; the rest presumably lies in functional status, cognition, nutrition and the social circumstances of discharge, none of which we recorded. It should also be borne in mind that these multivariable estimates derive from the 487 operated patients with complete data rather than from the whole cohort of 624 patients, and that this complete-case selection may itself have introduced bias, as discussed in the Limitations. Comparison with a published series is instructive. Our median of 10 days is close to the 9 to 10 days reported after orthogeriatric reorganization in France [22] but well short of the 21 to 27 days described in the British units that first reported integrated care [23], so room for improvement lies more before the operation rather than after it, with the postoperative phase already being short at a median of 6 days. Comprehensive geriatric assessment and individualized rehabilitation remain the appropriate pathway for patients flagged as high-risk [19,20,21]; what our data suggests is that a simple comorbidity count may be a practical admission-time trigger for that assessment, or at least useful as a weighted index for this purpose, although this requires confirmation in other cohorts.

## 5. Limitations of the Study

Several limitations qualify these findings. The design is retrospective and single-center, so the estimated coefficients reflect the case mix and the operative logistics of one service and should not be transported directly to settings with different theater access. The outcome is strongly right-skewed (skewness 5.02), and although we addressed this by log-transforming LOS and using rank-based tests, several associations reported in earlier work on this kind of data, including those of age and hemoglobin, do not survive that treatment; readers comparing our results with parametric analyses elsewhere should bear this in mind. The interval to surgery is a component of total LOS, so estimates against that outcome are partly structural; we therefore report postoperative stay as a secondary outcome, but this restricts those analyses to operated patients and the corresponding models describe the 487 operated patients with complete data rather than the whole cohort. Missing values were not imputed, and the multivariable analyses therefore rest on a complete-case sample of 487 patients, 78.0% of the original cohort of 624. This complete-case selection may have introduced selection bias. The second perioperative hemoglobin outcome was absent in 106 of 624 records (17.0%), and its absence is unlikely to have been random: a repeat determination is more readily omitted in patients with an uncomplicated course who are discharged early, and it cannot be obtained in patients who die before surgery, so missingness is plausibly related to disease severity and to LOS itself. The 96 non-operated patients, for whom the operative interval is undefined, are a group that in this population typically includes those least fit for anesthesia, and their exclusion is likely to have removed the frailest end of the spectrum from the models. The regression coefficients therefore describe operated patients with a documented perioperative course; if missingness was associated with patient characteristics, disease severity, or LOS, the estimates may be biased in a direction that cannot be determined from these data, and the comparison between comorbidity measures is subject to the same caveat. Multiple imputation or inverse-probability weighting in a prospectively collected dataset would be needed to address this. All analyses are exploratory and no correction for multiplicity was applied, so borderline *p* values should be interpreted accordingly. The CCI was reconstructed from broad comorbidity categories, and although we derived a severity-graded version from the admission laboratory data, this grading relies on a single creatinine measure in an acutely injured patient and cannot distinguish chronic kidney disease from acute kidney injury, nor capture the severity of liver disease, the metastatic status of malignancy or the functional class of heart failure. The ACCI is by construction a function of the CCI score and age, so the model containing both is collinear and the corresponding estimate should be read only as a comparison of measures. Pre-fracture functional status, cognitive status, nutritional markers and the social circumstances governing discharge were not available and are likely to account for a substantial share of the unexplained variance. Because death both competes with discharge and truncates the recorded stay, mortality could not be modeled as a conventional predictor, and the shorter stay of patients who died without surgery should be interpreted in that light. No a priori sample size calculation was performed. Finally, the association between preoperative delay and LOS is observational: patients who waited longer may have waited because they were less fit for surgery, so the causal direction cannot be established from these data and would require adjustment for anesthetic risk or a prospective design.

## 6. Conclusions

In this cohort of 624 older adults hospitalized for hip fracture, the number of comorbidities was the only patient characteristic consistently associated with LOS in both univariate and multivariable analysis and for both total and postoperative stay. The perioperative fall in hemoglobin contributed in the multivariable models but not for rank correlation. Age, sex, fracture type, area of residence and the CCI in all three forms showed no robust association; the apparent effects of age and sex reported on parametric tests did not survive analysis appropriate to a markedly skewed outcome. The interval to surgery dominated total stay, but this is largely because the waiting time is itself part of the admission: its effect on postoperative stay was small and not statistically significant.

Two implications follow for the organization of care. The first concerns the preoperative interval. A median wait of 4 days, with only a quarter of patients operated on within 48 h, represents avoidable bed-days regardless of what happens afterwards, and shortening this is the change most directly under institutional control. The second concerns risk stratification. In our data a weighted comorbidity score did not improve the prediction of stay: the CCI, tested in unweighted, age-adjusted and severity-graded forms, was not associated with LOS, whereas the simple count of active diagnoses was, although the gain in model fit was modest. This finding should be regarded as hypothesis-generating; it does not establish that the comorbidity count should replace the CCI, and such a substitution would require validation in independent cohorts and in healthcare settings with a different case mix and different operative logistics.

Given that the early identification of patients at risk of prolonged hospitalization is essential for optimizing the care pathway, geriatric assessment should be initiated at the time of hospital admission. At this stage, the number of active comorbidities is the indicator that is both readily available and consistently associated with hospital stay in our data, and it may serve as a simple admission-based screening tool to identify vulnerable patients who could benefit from an additional comprehensive geriatric assessment and to help prioritize geriatric interventions. However, the initial assessment should be multidimensional and should encompass not only comorbidity burden but also pre-fracture functional status, cognitive function, and nutritional status. Such an early approach would facilitate the identification, within the first hours of admission, of individual needs for medical optimization, complication prevention, and early planning of rehabilitation and discharge. Future prospective studies should evaluate the prognostic value of these components of comprehensive geriatric assessment and determine whether their systematic implementation at admission can reduce hospital LOS and improve clinical outcomes.

## Figures and Tables

**Table 1:** Epidemiological and clinical characteristics of the patients in the study group (*n* = 624).

Characteristic	*n*	%
*Sex*		
Female	451	72.3
Male	173	27.7
*Age (years)*		
Mean ± SD	79.73 ± 7.79	—
Median (min–max)	80 (65–101)	—
<80	307	49.2
≥80	317	50.8
*Area of residence*		
Urban	321	51.4
Rural	303	48.6
*Fracture type*		
Trochanteric fracture of the femur	396	63.5
Femoral neck fracture	228	36.5
*Comorbidities*		
Cardiovascular diseases	542	86.9
Anemic syndrome	209	33.5
Psychiatric disorders	124	19.9
Kidney diseases	107	17.1
Neurological diseases	102	16.3
Diabetes mellitus	100	16.0
Obesity	40	6.4
Malignancies	34	5.4
Liver diseases	23	3.7
Charlson Comorbidity Index, mean ± SD	1.68 ± 1.19	—
Charlson Comorbidity Index, median (min–max)	1 (0–7)	—
Charlson Comorbidity Index ≥ 3	149	23.9
Age-adjusted Charlson Comorbidity Index, mean ± SD	5.07 ± 1.40	—
*Management*		
Surgical intervention	528	84.6
Transfusion	12	1.9
*Outcomes*		
Length of stay (days), median (min–max)	10 (1–95)	—
Interval to surgery (days), median (min–max)	4 (0–18)	—
Postoperative death	22	3.5
Death without surgery	11	1.8

SD, standard deviation.

**Table 2:** Length of hospital stay (days) by demographic, fracture, and management characteristics.

Group	*n*	Median (IQR)	Mean ± SD	Min–Max	*p* (Mann–Whitney)	*p* (Welch)	*p* (ANOVA)
Total	624	10 (8–13)	10.62 ± 6.56	1–95	—	—	—
Male	173	10 (7–13)	11.45 ± 9.49	1–95	0.514	0.129	0.049
Female	451	10 (8–12)	10.29 ± 4.98	1–55			
Urban	321	10 (8–13)	10.65 ± 5.55	1–50	0.396	0.880	0.879
Rural	303	10 (8–12)	10.57 ± 7.50	1–95			
Trochanteric fracture	396	10 (8–12)	10.58 ± 5.65	1–55	0.835	0.856	0.843
Femoral neck fracture	228	10 (7–13)	10.68 ± 7.91	1–95			
Surgery	528	10 (8–13)	11.59 ± 6.49	3–95	<0.001	<0.001	<0.001
No surgery	96	4 (3–6)	5.25 ± 3.78	1–22			
Postoperative death	22	17.5 (13–25)	18.00 ± 9.15	4–34	<0.001	<0.001	<0.001
No postoperative death	602	10 (8–12)	10.35 ± 6.30	1–95			
Death without surgery	11	6 (4–10)	7.55 ± 4.85	1–17	0.045	0.060	0.118
No death without surgery	613	10 (8–13)	10.67 ± 6.58	1–95			

IQR, interquartile range; SD, standard deviation. The Mann–Whitney U test is the primary comparison; Welch’s *t*-test and the one-way ANOVA on the untransformed scale are shown for comparability. Variances were unequal for sex (Levene’s test, *p* < 0.001) and for postoperative death (Levene’s test, *p* < 0.001).

**Table 3:** Sequential multivariable linear regression models for log-transformed length of hospital stay (*n* = 487 operated patients with complete data).

Model	Predictor Added	R	R^2^	Adj. R^2^	SE	F (Model)	*p* (Model)	ΔR^2^	F Change	*p* (Change)
1	Age	0.108	0.012	0.010	0.375	5.78	0.017	0.012	5.78	0.017
2	HbT1	0.114	0.013	0.009	0.375	3.19	0.042	0.001	0.61	0.436
3	HbT2	0.149	0.022	0.016	0.374	3.66	0.013	0.009	4.54	0.034
4	No. of comorbidities	0.283	0.080	0.072	0.363	10.45	<0.001	0.058	30.18	<0.001
5	Interval to surgery	0.626	0.391	0.385	0.295	61.89	<0.001	0.312	246.34	<0.001

SE, standard error of the estimate; ΔR^2^, change in R^2^ on adding the predictor. F (model) tests the whole model at that step; changes in F test only the predictor added. The two are frequently confused: at Model 5, Model F is 61.89, while the F change for the interval to surgery is 246.34. Predictors were pre-specified and entered in the order shown; no automated selection was used. HbT1, hemoglobin at admission; HbT2, hemoglobin at the second perioperative determination. R, multiple correlation coefficient; R^2^, coefficient of determination; Adj. R^2^, adjusted coefficient of determination; *p* (Model), *p* value of the whole model; *p* (Change), *p* value of the F change.

**Table 4:** Length of hospital stay (days) by Charlson Comorbidity Index stratum.

Charlson Stratum	*n*	Median (IQR)	Mean ± SD	Min–Max	*p* (Kruskal–Wallis)	*p* (ANOVA)
0–1	362	10 (8–12.75)	10.69 ± 6.89	1–95	0.169	0.184
2–3	208	10 (7–12)	10.13 ± 5.68	1–50		
≥4	54	11 (8–13)	11.94 ± 7.37	2–44		

IQR, interquartile range; SD, standard deviation. Kruskal–Wallis is the primary test; one-way ANOVA on the untransformed scale is shown for comparability (*n* = 624).

**Table 5:** Comparison of comorbidity and severity measures as predictors of length of hospital stay.

Measure	rho with LOS	*p*	Adj. R^2^ (Log LOS)	*p* of Term
Number of comorbidities (unweighted count)	0.119	0.003	0.385	<0.001
Charlson Comorbidity Index	−0.032	0.418	0.365	0.623
Age-adjusted Charlson Comorbidity Index	−0.028	0.491	0.365	0.516
Severity-graded Charlson Comorbidity Index	−0.031	0.432	0.366	0.268
eGFR (CKD-EPI 2021)	−0.064	0.113	0.366	0.273
Acute laboratory derangement score	0.043	0.289	0.367	0.130

LOS, length of stay; eGFR, estimated glomerular filtration rate; rho, Spearman’s rank correlation. Correlations were computed on all available records (*n* = 601 to 624 depending on the measure). Each adjusted R^2^ comes from a model of log-transformed LOS containing age, HbT1, HbT2, the interval to surgery and the measure listed, fitted on the common complete-case sample of operated patients (*n* = 487). Adj. R^2^, adjusted coefficient of determination.

## Data Availability

The original contributions presented in this study are included within the article. Further inquiries can be directed to the corresponding author.
